# Branched-chain amino acids, mitochondrial biogenesis, and healthspan: an evolutionary perspective

**DOI:** 10.18632/aging.100322

**Published:** 2011-04-30

**Authors:** Alessandra Valerio, Giuseppe D'Antona, Enzo Nisoli

**Affiliations:** ^1^ Pharmacology Unit, Department of Biomedical Sciences and Biotechnologies, Brescia University, Brescia 25123, Italy; ^2^ Department of Physiology, Human Physiology Unit and Interuniversity Institute of Myology, Pavia University, Pavia 27100, Italy; ^3^ Center for Study and Research on Obesity, Department of Pharmacology, Chemotherapy and Medical Toxicology, School of Medicine, Milan University, Milan 20129, Italy

**Keywords:** branched-chain amino acids, calorie restriction, aging, lifespan, nitric oxide, mitochondrial biogenesis, mammalian target of rapamycin

## Abstract

Malnutrition is common among older persons, with important consequences increasing frailty and morbidity and reducing health expectancy. On the contrary, calorie restriction (CR, a low-calorie dietary regimen with adequate nutrition) slows the progression of age-related diseases and extends the lifespan of many species. Identification of strategies mimicking key CR mechanisms – increased mitochondrial respiration and reduced production of oxygen radicals – is a hot topic in gerontology. Dietary supplementation with essential and/or branched chain amino acids (BCAAs) exerts a variety of beneficial effects in experimental animals and humans and has been recently demonstrated to support cardiac and skeletal muscle mitochondrial biogenesis, prevent oxidative damage, and enhance physical endurance in middle-aged mice, resulting in prolonged survival. Here we review recent studies addressing the possible role of BCAAs in energy metabolism and in the longevity of species ranging from unicellular organisms to mammals. We also summarize observations from human studies supporting the exciting hypothesis that dietary BCAA enriched mixture supplementation might be a health-promoting strategy in aged patients at risk.

## INTRODUCTION

At an age when proper nutrition is a fundamental health requirement, almost half the elderly people in developed countries are not adequately nourished. By the widely used Mini Nutritional Assessment, the reported prevalence of nutritional risk in older subjects is approximately 45% in the community, with higher values in domiciliary care settings or hospitals and 84% to 100% in residential care facilities [1]. Malnutrition is defined as a state in which a deficiency, excess or imbalance of energy, protein and other nutrients causes adverse effects on body form, function and clinical outcome [2]. Excess caloric intake or consumption of palatable energy-dense foods increases fat accumulation and vulnerability to a range of age-related diseases, including type 2 diabetes, cardiovascular disorders, and cancer. A substantial number of older people in westernized countries are overweight. Although the increase in the relative risk for death that is associated with being obese is not as great in older subjects as it is in young adults, functional capacity, mobility and quality of life are significantly reduced in the obese elderly [3]. On the other side, undernutrition is a common feature among aged individuals, due to multiple reasons that include reduced appetite and food intake – the physiologic ‘‘anorexia of aging’’ – and numerous nonphysiologic factors, i.e., impaired nutrient absorption and other age-related medical, psychological and social changes [1, 2]. Inadequate nutritional intake may conduct to a global functional decline referred to as frailty, a newly recognized geriatric syndrome due to excess demand imposed upon reduced capacity [4]. Particularly, potein-energy undernutrition is associated with reduced strength, decreased bone mass, immune dysfunction, anemia, impaired cognitive function, poor wound healing, delayed recovering from surgery and higher hospitalization rate and is a strong independent predictor of mortality in elderly people [3].

Geriatric nutrition research aims to decipher the molecular mechanisms involved in the effects of dietary nutrients and to clarify their efficacy in the attainment of healthy aging. Several studies focused in particular on the effects of varying nutrient supply on animal and human longevity, with responses strongly dependent on genotype, age, nutrients, and regulation of nutrient-sensing pathways [5, 6].

Calorie restriction (CR), a low-calorie dietary regimen without malnutrition, decreases the incidence of several age-associated disorders and is considered the gold-standard, non-genetic approach for lifespan extension [7]. A body of evidence in several organisms demonstrates that an increase in mitochondrial activity, together with activation of the reactive oxigen species (ROS) defense system, is associated with the salutary effects of the CR regimen, [7, 8]. Although it has beneficial effects in humans [9], long-term CR requires a major commitment of will power and its possible disadvantages remain to be determined, particularly in the elderly [10]. Therefore, the concept of calorie restriction mimetics – that could provide the health benefits of CR without reduction in food intake – has become a hot area of investigation within gerontology [10].

Previous observations in yeast suggested that the branched-chain amino acids (BCAAs) leucine, isoleucine, and valine might be potential candidates in promoting survival [11]. We recently demonstrated that long-term dietary supplementation with a specific BCAA-enriched amino acid mixture (BCAAem) increased average lifespan of male mice [12]. This was accompanied by increased mitochondrial biogenesis and sirtuin 1 (SIRT1) expression and by up-regulated ROS defense system, with reduced oxidative damage, both in cardiac and skeletal muscles of middle aged mice [12]. The present article will analyse existing knowledge from various model organisms, from yeast to mammals, as well as from human studies, as a contribution to explore the possible effects of promoting mitochondrial function through BCAA supplementation on the health expectancy of aged subjects.

### Model organisms

#### Saccharomyces cerevisiea

The budding yeast, *Saccharomyces cerevisiae*, has been widely used for the identification of genes and cellular and biochemical pathways that affect the aging process. In unicellular yeast, aging mechanisms can be investigated by measuring replicative lifespan (RLS, the number of daughters produced by each dividing mother cell), or chronological lifespan (CLS, the capacity of stationary G_0_ cultures to maintain viability over time) [13]. CR, that is known to lengthen the mean and maximum lifespan of many species [7], extends both RLS and CLS [14-16]. The NAD^+^-dependent histone deacetylase, *Sir2*, a well-characterized RLS factor [17], is required for yeast RLS extension by CR [14]. Both yeast RLS and CLS are also affected by genetic interventions on lifespan effectors related to nutrient signaling, i.e., deletion of the yeast *Sch9* gene [which is homologous to the mammalian Akt/PKB implicated in the insulin-like growth factor (IGF) signaling] and mutations in the target of rapamycin (TOR) signaling pathway [18-20]. *Saccharomyces cerevisiae* is a facultative anaerobe that, under standard laboratory growth conditions (2% glucose), generates ATP largely by fermentation. Interestingly, deletion of the *TOR1* gene and CR cause a shift in glucose metabolism from fermentation – based on anaerobic glycolysis – toward respiration – based on oxidative metabolism involving the electron transport chain (ETC) – in both lifespan models [15, 20, 21], revealing a strong link between prolongevity effects and mitochondrial function.

In detail, moderate CR (modelled in yeast by reducing the glucose content of the media from 2% to 0.5%) leads to a 25% increase in the RLS together with increased transcription of respiratory genes and a higher rate of oxygen consumption [21]. Overexpression of the transcription factor *Hap4*, that causes a switch from fermentation to respiration, is sufficient to increase yeast RLS by 35% [21]. Gowth at 0.5% glucose fails to extend RLS in *cyt1* yeast mutants with impaired electron transport, suggesting that the metabolic shift toward respiration is necessary for lifespan extension mediated by CR [21]. This finding has been questioned by other studies, showing that increased respiration is not required for RLS extension by CR [22], in that CR at very low (0.05%) glucose concentrations increased lifespan in yeast strains lacking mtDNA (ρ_0_ cells) or in *cyt1* mutants [22]. However, the fact that alternate pathways promoting longevity are induced in yeast strains lacking respiratory capacity does not negate the role of mitochondrial respiratory function in CR-induced prolongevity when the organelles are functional [23]. An increase in genes involved in respiratory metabolism and mitochondrial function and an induction of tricarboxylic acid (TCA) cycle, at least partially due to *Hap4* up-regulation, has been recently reported to play a striking role in yeast CR lifespan extension models (0.1% glucose or elimination of nonessential amino acids) [24].

*Saccharomyces cerevisiae* has also served as a model organism to investigate the role of amino acid homeostasis in aging. It has been found that low levels of essential amino acids reduce CLS. Further, CLS has been recently studied in yeast grown in media supplemented with different amino acids. Increased availability of leucine, isoleucine and valine extended CLS and reduced the expression of GCN4, a transcriptional regulator of general amino acid control pathway, which regulates cellular amino acid homeostasis at a global level [11]. Conversely, the amino acid-mediated CLS extension was suppressed by constitutive overexpression of GCN4 [11]. The fact that leucine, isoleucine and valine were most important for CLS points to a special status for the BCAAs during aging. Accordingly, deletion of LEU3, a zinc finger transcription factor involved in BCAAs synthesis, dramatically increased CLS in the absence of amino acid supplements.

#### Caenorhabditis elegans

The nematode *Caenorhabditis elegans (C. elegans)* normally has a lifespan of about three weeks. The dauer larva, however, lives several times longer. In addition, several *C. elegans* mutants have increased longevity [25].

Surprisingly, a large class of*C. elegans* mutants with either genetic or RNA interference (RNAi)-mediated disruptions in genes essential for the function of mitochondrial ETC – the so-called Mit mutants – are long-lived (see [23] for review). The *isp-1* mutant bears a missense mutation in the Rieske iron sulphur protein (ISP), a subunit of the mitochondrial complex III. This mutant shows decreased mitochondrial respiration, low oxygen consumption, and prolonged lifespan [26]. Also the *clk-1* mutants, with defective ubiquinone (UQ_9_), the electron acceptor in complex I-dependent respiration, have increased lifespan [27]. The underlying cause for the increased lifespan of *clk-1* is a point of debate. In *clk-1* mutants, a ubiquinone intermediate (demethoxyubiquinone, DMQ_9_) accumulates to functionally replace ubiquinone, so that *clk-1* worms respire almost normally and show ATP levels unchanged or even higher than those of the wild type strain, strongly implying that their longevity is not the direct consequence of decreased energy metabolism [27]. Others have proposed that, despite a specific defect in complex I-dependent respiration and equal or increased ROS production, mitochondria of*clk-1* mutants scavenge ROS more effectively than wild type due to the presence of DMQ_9_, leading to reduced oxidative damage [28]. It has to be noticed that not all mutations that disrupt the ETC in *C. elegans* lead to an increase in lifespan. The *gas-1* mutants are characterized by low complex I-specific respiration, intense oxidative damage in mitochondrial proteins and very short lifespan [28]. The *mev-1(kn-1)* mutant, with a deletion in a subunit of complex II has a shortened lifespan [29]. Moreover, deletion of *phb-1* or *phb-2* (coding for mitochondrial prohibitins) has been found to influence ATP levels, animal fat content, mitochondrial proliferation and lifespan in a genetic background- and age-specific manner [30].

Prolongevity effects of CR have been described in *C. elegans* models. Up-regulated or unchanged metabolic rate, respectively, have been initially described in long-lived *eat* mutants (having a feeding defect) and calorie restricted worms [31]. More recently, it has been demonstrated that CR-mediated *C. elegans* lifespan extension requires an increase in the respiration rate (whole-body oxygen consumption) [32]. Accordingly, specific restriction of intracellular glucose by 2-deoxy-glucose treatment also extends lifespan in worms by promoting mitochondrial respiration and an antioxidant response [33].

The latest way to investigate long-lived worms distinctive features is metabolite profiling, also called metabolomics. By this approach, Fuchs and coworkers [25] simultaneously studied different models of long-lived worms, i.e., dauer larvae, several *daf-2* mutants [affecting the insulin/IGF-1 (IIS) signaling pathway], and *ife-2* mutants (with disrupted eukaryotic translation initiation factor, eIF4E). The metabolic responses of all these mutants were similar, allowing to identify a “metabolic signature” of long-life in worms. The most striking response was the up-regulation of the BCAAs isoleucine, leucine and valine in long-lived *daf-2* and *ife-2* mutants. The longevity prolonging effects of DAF-2/IIS pathway suppression is mediated by the activation of the FOXO transcription factor DAF-16. To find out whether the metabolite changes were also DAF-16 dependent, metabolic profiling of wild type worms was compared to that of the *daf-2* mutants, the *daf-16* mutants, or double-mutant worms. Of interest, isoleucine, valine, and leucine changes showed the classic pattern of DAF-16 dependence, making BCAAs strong candidates for having a causal role in long life [25]. A more recent metabolomic study brought further evidence that BCAA levels are increased in a DAF-16-dependent manner in long-living *daf-2* mutants [34]. Like other animals, *C. elegans* cannot synthesize BCAAs, so that their levels depend on changes in protein turnover or in BCAAs catabolism. Key regulators of BCAA catabolic pathway are BCAA aminotransferase (BCAT) and the branched-chain α-ketoacid dehydrogenase (BCKDH) complex [35]. It has been hypothesized that the down-regulation of genes encoding for the BCKDH complex might be responsible for the accumulation of BCAAs in long-lived worms [25]. Altered transcript levels of various genes involved in BCAA metabolism have been found in long-lived mutants by other authors[34]. Although strong BCKDH inactivation causes severe embryonic and larval phenotypes in *C. elegans* and maple syrup urine disease in humans, it has been suggested that partial down-regulation of the BCKDH complex or subtle elevation of BCAA levels by diet might confer long life [25].

#### Drosophila melanogaster

The fruit fly *Drosophila melanogaster* has a relatively short lifespan and has been extensively used as a model organism for aging studies. Early reports revealed reduced mitochondrial number and mitochondrial structural changes in the aged *Drosophila*[36]. The expression levels of various transcription factors essential for mtDNA replication, including mitochondrial DNA transcription factor A (Tfam), are decreased in old flies. Further, aged flies have reduced transcripts of genes of the ETC and the TCA cycle and reduced ATP synthesis [37]. Conversely, the genes involved in oxidative phosphorylation are up-regulated in long-lived *Drosophila* overexpressing a small mitochondrial chaperone, Hsp22 [38].

Engineering fruit flies to overexpress a single-subunit mitochondrial respiratory complex from yeast showed tissue-specific effects on longevity. Overexpression of the NADH-ubiquinone oxidoreductase (NDI1) of *Saccharomyces cerevisiae* in the adipose tissue of the fruit fly was found to exert a negative impact on longevity, while neuronal NDI1 overexpression resulted in life extension [39]. Ubiquitous expression of NDI1 significantly increased fly longevity [40], supporting the idea that increased respiration can retard the *Drosophila* aging process. Increased mitochondrial activity plays also a causative role in CR-mediated extension of *Drosophila* lifespan, since knock-down of either complex I or IV subunits leads to diminished lifespan extension under CR [41].

Mitochondria are critical in providing metabolites for the *de novo* synthesis of nonessential amino acids. *Drosophila* larvae grown in low yeast food, thus on amino acid starvation, showed strongly reduced mitochondrial abundance, mitochondrial respiratory proteins and respiration activity in larval fat body, the fly adipose/liver tissue [42]. This correlated with reduced expression of enzymes involved in glutamine metabolism [42], strongly suggesting that the amino acid metabolism is coordinated with mitochondrial abundance and activity. The *Drosophila* transcription factor *Delg* was proposed to coordinate mitochondrial functions according to nutrient availability, and to adjust the synthesis of nonessential amino acids to the uptake of essential amino acids [42].

#### Mus musculus

Despite the interest of results obtained in lower organisms, use of mammalian models, such as mice, is likely to be more relevant for understanding the aging-related processes that occur in humans. Naturally long-lived mouse mutants and various genetically altered mice with extended lifespan have been studied [43]. A body of evidence indicated the GH/IGF-1 axis as a major contributor to longevity effects in mice. Further evidence implicated increased capacity to resist oxidative damage in mice survival [43], particularly thanks to studies on p66(*Shc*), a crucial regulator of ROS levels whose deletion in mice prolongs lifespan (See Trinei et al. [44] for recent review) Efficient renewal of functional mitochondria is known to reduce mitochondrial ROS production [7]. Interestingly, mutations affecting GH/IGF-1 signaling induce mitochondrial gene expression and oxidative metabolism in mice [45-46].

The life-extending effects of the CR regimen in rodents are well known [7]. We first demonstrated that CR, by feeding mice on alternate days, promotes mitochondrial renewal in several tissues, mainly by increasing the expression of peroxisome proliferator-activated receptor γ coactivator 1α (PGC-1α) [8], a powerful regulator of mitochondrial biogenesis and of the reactive ROS defense system [47]. CR also induced the expression of endothelial nitric oxide synthase (eNOS) and SIRT1, the mammalian orthologue of the yeast *Sir2* gene linked to lifespan extension, enhanced mitochondrial biogenesis and decreased ROS production [8]. Our observations have been subsequently confirmed by others [48, 49], also in humans [50]. The CR effects were blunted in eNOS-null mutant (eNOS^-/-^) mice [8]. Interestingly, eNOS^-/-^ mice have defective mitochondrial biogenesis, reduced SIRT1 expression [8, 51, 52] and display metabolic derangements, age-related diseases and shortened lifespan [53, 54].

The relevance of boosting mitochondrial function to preserve mammalian health and longevity has been recently proved by Safdar et al. [55]. In a strain of mice prone to mtDNA damage and with reduced lifespan (i.e., the mtDNA mutator mouse, designated the PolG mouse, a model of progeroid aging that exhibits elevated mtDNA point mutations), a regime of endurance training induced mitochondrial biogenesis, increased mitochondrial respiratory capacity, and prevented mtDNA damage. Furthermore, the trained mice no longer exhibited premature mortality or other symptoms associated with accellerated aging, including fat loss, muscle loss, anemia, and graying fur.

Three of the seven mammalian sirtuins (SIRT3, SIRT4, and SIRT5) are targeted to mitochondria and can their expression be differently modulated by the CR regimen [56-58]. Studies in SIRT3 and SIRT5 mutant mice, that are prone to age-related disorders [59] have recently provided unexpected links among CR-related mitochondrial changes and amino acid metabolism (see below).

### Antiaging effects of dietary BCAA supplementation in mice

In search for CR-mimetic compounds, we recently investigated the effects of a balanced amino acid mixture with a high content of branched-chain and other essential amino acids (BCAA-enriched mixture, BCAAem; % composition: leucine 31.3, lysine 16.2, isoleucine 15.6, valine 15.6, threonine 8.8, cysteine 3.8, histidine 3.8, phenylalanine 2.5, methionine 1.3, tyrosine 0.7, tryptophan 0.5) which had been found to improve age-related disorders in animals and humans (see below). We demonstrated that BCAAem oral supplementation (1.5 mg/g body weight/day beginning at 9 months) increases the average, but not maximal lifespan of male mice [12]. Along with increased survival, BCAAem-supplemented middle-aged (16 months) mice showed up-regulated PGC-1α and SIRT1 expression and enhanced mitochondrial biogenesis and function in cardiac and skeletal muscles but not in adipose tissue or liver. Further, the BCAAem preserved muscle fiber size and improved physical endurance and motor coordination in middle-aged mice [12]. Notably, BCAAem was unable to affect muscle mitochondrial density and function and failed to extend average lifespan in eNOS^-/-^ mice. The prolonged survival due to BCAAem supplementation was also associated with increased expression of genes involved in antioxidant defense and marked reduction of ROS production in cardiac and skeletal muscles of wild type but not eNOS^-/-^ mice. Of interest, BCAAem-mediated effects were even more remarkable in long-term exercise-trained (running 30 to 60 min 5 days/week for 4 weeks) middle-aged mice. In young animals (4-6 months old), the mixture was ineffective.

Which mechanisms are involved in the observed BCAAem effects? mTOR complex 1 (mTORC1; mammalian TOR [mTOR] in complex with raptor) is a key regulator of protein synthesis and cell growth in response to nutrient amino acids. BCAAs increase mTORC1 activity [60], which favours cell oxidative capacity [61] and PGC-1α-mediated mitochondrial gene expression [62]. We found that BCAAem activated mTOR and its downstream signals and that the mTORC1 inhibitor rapamycin antagonized the mitochondrial biogenesis effects of BCAAem in cardiomyocytes [12]. We also found evidence suggesting that BCAAem-activated mTOR signaling might enhance mitochondrial biogenesis partly through increasing the NO generating system. Moreover, eNOS gene silencing decreased the mTOR activation by BCAAem in cells and BCAAem supplementation was unable to activate mTOR signaling in eNOS^-/-^ mice [12]. Thus, a positive feedback mechanism between eNOS and mTOR could promote the BCAAem effects. How amino acids influence and activate mTORC1 was not been well delineated until a most recent study, which established inositol polyphosphate multikinase (IPMK) as a key determinant of leucine- or total amino acid-mediated signaling to mTORC1 in mice [63]. Amino acid-stimulated mTOR activation occurs independently of IPMK's catalytic activity. Instead, IPMK acts by stabilizing the mTOR-raptor association in the mTORC1 complex [63].

We did not specifically investigate the contribution of enhanced mTOR signaling in BCAAem-mediated increase of mice average survival. Interestingly, selective knockout of either mTOR or the mTORC1 component raptor in skeletal muscle decreased oxidative capacity, mitochondrial gene expression, and survival [64, 65]. However, reduced TOR signaling is thought to be a putative mechanism mediating lifespan extension by CR (see [66] for review). Mice with deletion of the mTOR substrate ribosomal S6 protein kinase (S6K) have increased lifespan [67]. Further, chronic rapamycin treatment in mice exerts prolongevity effects [68], yet this finding does not conclusively prove that mTOR inhibition is the mechanism involved in rapamycin-mediated life extension. Notably, rapamycin was unable to increase *Drosophila* lifespan [69]. Moreover, mTOR inhibition-mediated lifespan extension displays a gender effect clearly distinguishable from CR. Unlike CR, rapamycin is more efficacious in female than in male mice [68], while S6K deletion increases lifespan only in females but not in males [67]. The gender-specific pattern of mTOR inhibition in aged individuals remains a problem to be solved [70]. Again, the role of mTOR in CR is tissue specific. CR reduces mTOR signaling in liver [71] but increases it in WAT and heart [72]. Further, the CR-mediated increase of mitochondrial function in different tissues [8] is not consistent with reduced mTOR signaling. In addition, recent evidence indicates that mTOR signaling is down- or up-regulated depending of age and the type of CR regimen [73]. All in all, the role of mTOR in CR mechanisms is complex and not yet conclusively clarified [74]. With this in mind, more work needs to be done to address the possible contribution of mTOR in BCAAem prolongevity effects.

**Figure 1.**
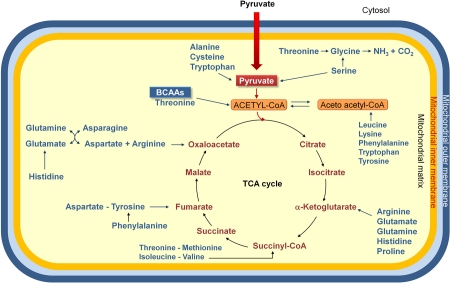
Amino acids are degraded to compounds that can be metabolized to CO_2_ and H_2_O, or used in gluconeogenesis. Indeed the oxidative degradation of amino acids produces 10 – 15% of total metabolic energy in animals. The standard amino acids are degraded to one among the seven metabolic intermediates (pyruvate, α-ketoglutarate, succinyl-CoA, fumarate, oxaloacetate, acetyl-CoA or acetoacetate). Thus, amino acids may be divided into two groups, on the basis of their catabolic pathways: 1) gluconeogenic amino acids, which are catabolized to pyruvate, α-ketoglutarate, succinyl-CoA, fumarate or oxaloacetate, and are glucose precursors; 2) ketogenic amino acids, which are catabolized to acetyl-CoA or acetoacetate, and, thus, may be transformed into fatty acids or ketone bodies. Some amino acids are precursors both of carbohydrates and ketone bodies. Because mammals have no metabolic pathway which allows a net transformation of acetyl-CoA or acetoacetate to gluconeogenic precursors, no net synthesis of carbohydrates is possible from lysine and leucine, exclusively ketogenic amino acids. BCAAs, branched-chain amino acids.

**Figure 2.**
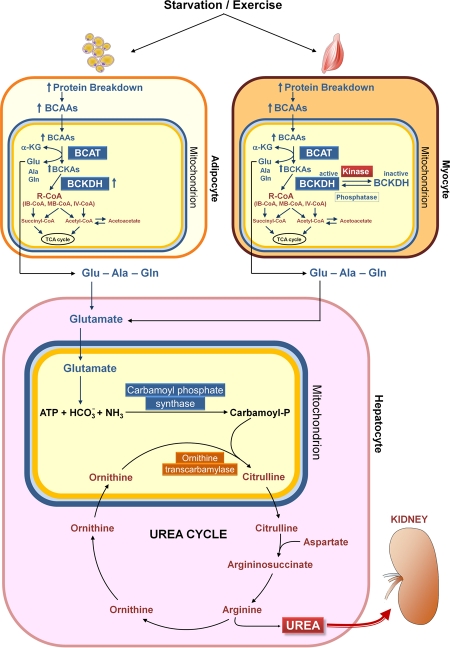
Catabolism of branched-chain amino acids. Starvation and exercise stimulate protein breakdown, thereby increasing the concentrations of branched-chain amino acids (BCAAs) in adipose and muscle cells. The BCAAs are transaminated in muscle mitochondria by branched-chain aminotransferase (BCAT), and branched-chain α-keto acids (BCKAs, especially α-keto acid from leucine) inhibit branched-chain α-keto acid dehydrogenase kinase, resulting in elevation of the active state of the rate limiting enzyme branched-chain α-keto acid dehydrogenase complex (BCKDH). Recent results indicate a novel mechanism for regulation of BCAA oxidation in adipose tissue, i.e. changes in the expression of BCAA enzymes, in contrast to altered BCKDH phosphorylation, which is the major mechanism regulating BCAA oxidation in muscle and liver [121]. Ala, alanine; α-KG, α-ketoglutarate; Glu, glutamate; Gln, glutamine; IB-CoA, isobutyryl-coenzyme A; IV-CoA, isovaleryl-coenzyme A; MB-CoA, α-methylbutyryl-coenzyme A; R-CoA, acyl-coenzyme A; TCA, tricarboxylic acid.

Why does the BCAAem promote mitochondrial biogenesis in metabolically active tissues and what is the relationship between this effect and the CR-induced changes in mitochondrial function? Conclusive answers are not available yet, but a sound hypothesis can be put forward. First, amino acids are important precursors of TCA cycle components (Fig. 1). Secondly, amino acid catabolism leads to production of ammonia, which is metabolized via the urea cycle, whose first two steps occurr in the mitochondrial matrix (Fig. 2). Thus, the amino acid supplementation could induce mitochondrial biogenesis to promote catabolism of amino acid themselves. Interestingly, Nakagawa et al. [58] demonstrated that during long-term CR or a high protein diet, the mitochondrial SIRT5 deacetylates and activates carbamoyl phosphate synthase 1, the first and regulated step of urea cycle. Accordingly, Hallows et al. [59] have more recently demonstrated that the mitochondrial deacetylase SIRT3 directly regulates ornithine transcarbamoylase activity, the second step of the urea cycle, thus promoting the amino acid catabolism during CR. These findings suggest that the amino acid-induced mitochondrial biogenesis might be functional to amino acid catabolism and that amino acids might be, either directly or indirectly, related to the effects of CR on survival of mammals.

### Health effects of BCAAs in mammals

For decades, dietary supplementation with amino acids has been proposed in various physiological or pathological conditions. Based on the recent progress in our understanding of the BCAA cell signaling and *in vivo* metabolism, and on accumulating experimental results, the concept that dietary BCAA supplementation might have health effects is now experiencing a major revival (see [75] for review). Latest evidence from basic and clinical studies might extend the use of specific amino acid mixtures for the prevention and/or treatment of diverse human disorders.

#### Animal studies: an update

The effects of BCAA intake have been investigated in a number of disease models, including obesity and metabolic disorders, liver disease, impaired immunity, muscle atrophy, cancer, and a variety of injury (postoperative, trauma, burn, and sepsis) [75]. Here we will briefly revise the most recent developments of this topic.

First of all, BCAAs appear to have unique obesity-related effects. BCAAs, and in particular leucine, increase fat leptin secretion [76], decrease food intake and body weight via mTOR signaling [77], and improve muscle glucose uptake and whole body glucose metabolism [78]. However, obese rodents (*ob*/*ob* mice and *fa*/*fa* Zucker rats) exhibit elevated plasma BCAA levels [78]. To explore loss of catabolic capacity as a potential contributor to the obesity-related rises in BCAAs, She et al. [78] assessed possible changes in the first two enzymatic steps of BCAA catabolism, namely, BCAT and the BCKDH complex. They found tissue-specific alterations in BCAA catabolic enzymes, involving a decline of BCKDH E1α in liver and adipose tissue, but not in muscle, possibly contributing to the rise in plasma BCAAs in obesity. In a separate series of experiments, the same investigators generated mice in which the gene encoding the BCAT2 isozyme was disrupted [79]. They found that rises in plasma BCAAs were associated with improvements in glucose tolerance and resistance to diet-induced obesity in these animals. The authors proposed that increased protein synthesis and degradation would contribute directly to increased energy expenditure in mice lacking peripheral BCAA metabolism. These findings suggest that the increased BCAA levels in obese animals might be compensatory to obesogenic stimuli.

Actually, controversy exists about the effects of increasing dietary leucine on insulin sensitivity. For example, Zhang and colleagues have demonstrated that an increased leucine dietary intake improves the whole-body glucose metabolism in mice maintained on a high-fat diet [80]. By contrast, in a recent study, leucine deprivation was observed to increase whole-body insulin sensitivity [81]. Leucine deprivation improved hepatic insulin sensitivity by activating general control nonderepressible GCN2, decreasing mTOR/S6K1 and activating AMP-activated protein kinase (AMPK) signaling. Again, leucine deprivation improved insulin sensitivity under insulin-resistant conditions [81]. Noteworthy, Noguchi et al. [82] designed a novel diet with an elevated ratio of essential to nonessential amino acids (high-E/N diet). Dietary proteins in the high-E/N diet were partially replaced with a mixture of free ketogenic essential amino acids (leucine, isoleucine, valine, lysine and threonine) without altering dietary carbohydrate and fat content. This dietary amino acid manipulation improved glucose tolerance, decreased lipogenesis and prevented hepatic steatosis in diet-induced obese mice, and was suggested as a novel preventive and therapeutic approach for non-alcoholic fatty liver disease. Accordingly, in a recent study, rats orally administered an amino acid mixture (containing cysteine, methionine, valine, isoleucine and different concentrations of leucine) together with a high-glucose solution, have shown an improved glucose tolerance as compared to non-supplemented animals [83]. Overall, these results would suggest that specific mixtures of amino acids, rather than a single amino acid supplement, may be more efficacious in lowering the blood glucose response to a glucose challenge.

A promising area of preclinical research is regarding the effects of BCAAs on skeletal muscle atrophy. We observed that BCAAem intake preserves muscle fiber size and improved physical endurance and motor coordination in middle-aged mice [12]. Accordingly, an amino acid mixture with BCAAem composition has been found to improve sarcopenia, i.e., the aging-associated loss of muscle mass [84], an effect possibly due to the recovery of the altered Akt/mTOR signaling in muscles of aged rats [85].Correspondingly, other groups have recently reported that BCAAs decrease protein breakdown and protect against dexamethasone-induced soleus muscle atrophy in rats [86]. BCAAem-mediated improvement of muscle functional capacity was further enhanced by exercise training [12]. Exercise promotes longevity and is the best intervention to alleviate and reverse sarcopenia and frailty in the elderly [87]. It has been reported that concurrent intake of antioxidants (vitamin C and E) abolished some health-promoting effects of exercise in humans, by preventing the induction of the ROS sensors PGC-1α/β and consequent activation of ROS defense [88]. Our results suggest that the BCAAem could meet the need for a safe PGC-1α inducer in sarcopenia treatment [87] and a valid substitute for dietary supplementation with antioxidants in active elderly people.

The BCAAs leucine and valine have been also reported to prevent muscle atrophy in mice bearing a cachexia-inducing tumor [89]. Given the possible benefits of BCAAs in cancer patients, it would be of relevance to determine their effects on neoplastic cell growth. Dietary amino acids, incluning BCAAs, have been used in cancer models with mixed results [90-92]. Anyway, convincing data demonstrate that BCAA treatment does not directly potentiate neoplastic cell growth and may actually diminish neoplastic cell proliferation at supraphysiological concentrations [93]. Further investigation is needed to examine the effects of amino acid mixtures with different BCAA composition on normal and tumor cell proliferation.

Additional observations deal with the capability of the BCAAem formula to ameliorate myocardial dysfunction in diabetic rats [94] and to maintain the health of kidney in aged rats [95]. In particular, when administered orally at the beginning of rat senescence, BCAAem induces eNOS and vascular endothelial growth factor in the kidney, thus increasing vascularization and reducing kidney fibrosis. Improved vascularization and increased collagen deposition and fibroblast proliferation seem also to be involved in the cutaneous wound healing obtained with topical application of BCAAs and other essential amino acids in aged rats [96].

Again, BCAAs compete for large, neutral amino acid transport at the blood-brain barrier and can influence brain neurotrasmitter synthesis [97]. Experimental studies show that BCAAs have favourable effects on cognitive functions. BCAA supplementation has been reported to improve cognitive performance in active dogs, with greater benefit to senior dogs [98]. BCAA transamination plays an essential role in the synthesis of glutamate and subsequently of GABA. Cole et al. [99] evaluated mice subjected to traumatic brain injury, and found a significant reduction in BCAA concentration and neurotransmitter changes in the hippocampus. Dietary delivery of BCAAs to brain-injured mice restored hippocampal BCAA levels, synaptic glutamate and GABA pools and net synaptic efficacy, and eradicated injury-induced cognitive impairment [99].

#### Human studies: promising evidence

Emerging metabolomic technologies make it feasible to investigate the metabolic status of the whole human organism in high-throughput applications. Newgard et al. [100] studied subjects that become obese on a typical Western diet (with high fat and protein content). By metabolic profiling, they identified a cluster of obesity-related changes in specific amino acids that was associated with insulin resistance. In particular, circulating levels of the BCAAs were higher in obese compared to lean subjects [100]. Obesity was also associated with decreases in bioavailable IGF-1. The authors suggested that, in the context of overnutrition and low IGF-1 levels, circulating BCAAs rise, leading to an overload of BCAA catabolism that contributes to insulin resistance in obese subjects.

A more recent nested case-control study in the Framingham Offspring Study has investigated whether metabolite profiles could predict the development of type 2 diabetes [101]. Fasting concentrations of BCAAs and of two aromatic amino acids, phenylalanine and tyrosine, were found elevated up to 12 years before the onset of diabetes in high risk subjects as compared to propensity-matched control subjects. The strongest risk of future diabetes was associated to a combination of three amino acids, namely isoleucine, phenylalanine and tyrosine. In a more heterogeneous study sample, obtained by looking at a random set of controls from the Framingham cohort (having lower baseline body mass index and fasting glucose values compared to the case-control sample), the relative risk associated with elevated amino acids, though still significant, was attenuated [101]. The authors recognize that contrasting data exist on BCAA effects on glucose homeostasis and that further investigation is necessary to assess whether amino acids may be markers or effectors of insulin resistance.

On the other hand, sparse studies in wrestlers and in obese subjects have shown that BCAA supplementation exerts beneficial effects on body weight and body fat [102]. Most recently, the population-based International Study of Macro-/Micronutrients and Blood Pressure (INTERMAP) provided a unique opportunity to evaluate the effects of dietary BCAAs across different cultures. This high-quality study demonsrated that a higher BCAA intake is associated with a lower prevalence of being overweight or obese in middle-aged individuals from East Asian and Western countries [102]. In this line, Solerte et al. studied the effects of a balanced amino acid formula corresponding to the BCAAem in a long-term randomized study of elderly subjects with type 2 diabetes and found improved metabolic control (i.e., reduced glycated hemoglobin [HbA1c]) and insulin sensitivity [103]. Noteworthy, BCAAs effectively reduce insulin resistance in patients with chronic viral liver disease [104], and the health effects of BCAA supplementation in patients affected by liver disorders, including cirrhosis, was demons-trated in several reports [105-107].

A variety of amino acid mixtures have been used to restore the protein content of defective tissues, especially of skeletal muscles, in aged subjects [108, 109]. Dillon et al. [108] reported that 3-month supplementation with essential amino acids increases IGF-1 muscle levels and lean body mass in aged women, without affecting kidney function. The acute anabolic response to this supplementation (increased muscle protein fractional synthesis rate) was maintained over time, suggesting the possibility to improve skeletal muscle trophism in long-term treatment [108]. Various BCAA dietary supplements have been reported to reduce sarcopenia in elderly subjects. In a randomized trial involving 41 subjects with sarcopenia aged 66 to 84 years, intake of the BCAAem formula increased muscle mass, reduced tumor necrosis factor-α, and improved insulin sensitivity [110]. As a result, leucine-enriched balanced amino acid supplements are now considered as part of the nutritional recommendations for the management of sarcopenia [111].

Amino acid supplementation also inhibits inflammatory markers in chronic heart failure patients and might represent a promising therapeutic approach, particularly in the presence of the so-called wasting syndrome [112]. Accordingly, supplementation with the BCAAem formula improves exercise capacities in elderly subjects affected by chronic heart failure [113]. The latter effect was detectable also in aged individuals without evident disorders [114].

Certainly of interest are the recent reports that BCAAem intake reduces by 30% the incidence of infections acquired in geriatric long-term rehabilitation centers [115], increases the serum albumin and total proteins in hemodialysis patients, with reduction of inflammation markers and correction of anemia [116], and improves gas exchange and cognitive function score in patients with severe chronic obstructive pulmonary disease [117]. Equally important, in keeping with intriguing experimental data [99], parenteral supplementation of BCAAs was shown to enhance the cognitive recovery of patients with traumatic brain injury [118], even when on a vegetative or minimally conscious state [119].

### Conclusions and perspectives

**Figure 3.**
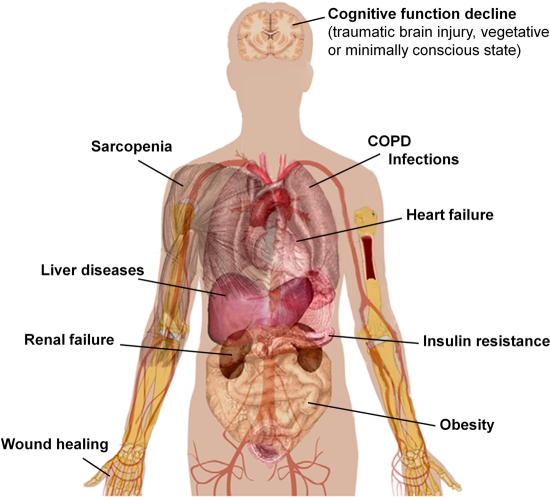
Possible health effects of amino acid mixtures in humans. Accurate clinical trials enrolling larger number of patients are necessary to confirm the safety and efficacy of BCAA/amino acid supplementation in geriatric patients. COPD, chronic obstructive pulmonary disease

A body of recent evidence suggest that amino acids, and in particular BCAAs, behave as evolutionary conserved modulators of lifespan of different organisms, ranging from yeast to mammals. Our data demonstrate that oral intake of a BCAA-enriched balanced amino acid mixture improves motor coordination and endurance and promotes longevity of male mice [12]. The key role of BCAAem on mitochondrial biogenesis, cell energy metabolism, and ROS scavenging systems, through the modulation of the mTOR/eNOS pathways, may explain most of the beneficial actions of this supplementation. Importantly, among the many genetic and pharmacological treatments that extend longevity in diverse animal models, BCAA supplementation has the add-on value to prolong animal health and functional capacities. Accordingly, likewise exercise, BCAAem does not affect maximum lifespan, but increases the median lifespan, an indicator that specific diseases have been prevented. Geriatricians have long recognized that disability, frailty, and age-related disease onset are critical issues that need to be addressed in older populations. Hence, the concept of healthspan has emerged as a key end point for geriatric studies to translate experimental findings into realistic clinical interventions [120]. A number of preclinical and clinical reports, here reviewed, supports the use of dietary supplementation with balanced amino acid formulas containing BCAAs to prevent disability and prolong healthy life expectancy of elderly subjects (see summary in Fig. 3).

A broad range of questions await answers. The first point to be clarified is the role that specific amino acid signatures can play, directly or indirectly, in the CR effects on healthspan. Next, taken into account the contradictory results that arise from leucine administration, there is need to investigate which amino acid (or specific amino acid combination) is required for the beneficial effects seen in mammals. Not last in importance, large, randomized clinical trials are necessary to assess the safety and efficacy of BCAA/amino acid supplementation for the prevention and treatment of the disabling consequences of energy depletion in the elderly.
